# Inducible pesticide tolerance in *Daphnia pulex* influenced by resource availability

**DOI:** 10.1002/ece3.4807

**Published:** 2019-01-10

**Authors:** Vanessa P. Wuerthner, Jared Jaeger, Paige S. Garramone, Connor O. Loomis, Yelena Pecheny, Rachel Reynolds, Lindsey Deluna, Samantha Klein, Michael Lam, Jessica Hua, George A. Meindl

**Affiliations:** ^1^ Biological Sciences Department Binghamton University (SUNY) Binghamton New York

**Keywords:** acetylcholine esterase inhibitor, carbamate, ecotoxicology, zooplankton

## Abstract

Pesticides are a ubiquitous contaminant in aquatic ecosystems. Despite the relative sensitivity of aquatic species to pesticides, growing evidence suggests that populations can respond to pesticides by evolving higher baseline tolerance or inducing a higher tolerance via phenotypic plasticity. While both mechanisms can allow organisms to persist when faced with pesticides, resource allocation theory suggests that tolerance may be related to resource acquisition by the organism. Using *Daphnia pulex*, we investigated how algal resource availability influenced the baseline and inducible tolerance of *D. pulex* to a carbamate insecticide, carbaryl. Individuals reared in high resource environments had a higher baseline carbaryl tolerance compared to those reared in low resource environments. However, *D. pulex *from low resource treatments exposed to sublethal concentrations of carbaryl early in development induced increased tolerance to a lethal concentration of carbaryl later in life. Only individuals reared in the low resource environment induced carbaryl tolerance. Collectively, this highlights the importance of considering resource availability in our understanding of pesticide tolerance.

## 1 INTRODUCTION

As human populations continue to expand, natural communities face selective pressures in the form of synthetic chemicals (Chevin, Lande, & Mace, 2010; Crispo et al., 2010; Georghiou, 1990; Hoffmann & Sgrò, 2011). Aquatic ecosystems, in particular, are subjected to chemical contamination from a variety of sources (Cheng, McCoy, & Grewal, 2014; McKnight, Rasmussen, Kronvang, Binning, & Bjerg, 2015). For example, modern agricultural practices simultaneously introduce synthetic pesticides and nutrient‐rich fertilizers into aquatic systems, both of which can negatively impact biological communities (Baker, Mudge, Thompson, Houlahan, & Kidd, 2016). While several organisms are known to respond to pesticide selection by evolving tolerance (Cothran, Brown, & Relyea, 2013; Georghiou, 1990; Jansen, Meester, Cielen, Buser, & Stoks, 2011), it is unclear how altered resource availability (e.g., cultural eutrophication) may limit or augment pesticide tolerance. Despite this uncertainty, whether species are able to adapt and persist in human‐altered environments likely depends on interactive effects between multiple environmental stressors (Blaustein et al., 2011; Coors & De Meester, 2008; Puccinelli, 2012).

In human‐modified environments, the elevated application rates of pesticides have led to the evolution of pesticide tolerance in both target and non‐target species (Brausch & Smith, 2009; Gilliom, 2007; Jansen, De Meester, et al., 2011; Stone, Gilliom, & Ryberg, 2014). Traditionally, tolerance to pesticides is predicted to arise over many generations via natural selection on constitutive traits resulting in organisms with high baseline tolerance to pesticides (Berg et al., 2010; Hoffmann & Sgrò, 2011; Lawrence et al., 2012). However, evidence suggests that phenotypic plasticity, defined as the capacity of a single genotype to produce different phenotypes in different environments, may be an alternative mechanism for organisms to more rapidly achieve tolerance to pesticides (Hua et al., 2015; Hua, Morehouse, & Relyea, 2013; Pigliucci, 2001; Schlichting, 2008; West‐Eberhard, 2003). Indeed, recent research demonstrated that some organisms reared in sublethal pesticide environments early in life can, within days, induce higher tolerance to pesticides via phenotypic plasticity (Hua et al., 2015; Jones & Relyea, 2015). The ability to rapidly induce tolerance may play a significant role in the persistence of aquatic organisms exposed to pesticides as many taxa are confined to the boundaries of the aquatic environment and cannot disperse to avoid exposure to contaminants (Moe et al., 2013). However, to date, the phenomenon of inducible tolerance to pesticides is known to occur only in 4 species: wood frogs, gray tree frogs, gulf killifish, and yellow‐fever mosquitos (Hua et al., 2015; Jones & Relyea, 2015; Oziolor, Howard, Lavado, & Matson, 2017; Poupardin et al., 2008). Given the ubiquitous nature of pesticide contamination in freshwater ecosystems, considering both constitutive and inducible mechanisms for tolerance is critical to developing a better understanding of whether and how natural communities will respond to contaminants (Stone et al., 2014).

Aquatic systems are complex and are affected by a variety of factors that may influence the mechanism by which organisms respond to pesticides (Barry, Logan, Ahokas, & Holdway, 1995; Pereira & Gonçalves, 2007; Pieters, Jager, Kraak, & Admiraal, 2006). For example, aquatic systems experience naturally occurring seasonal changes in nutrient inputs as well as rapid increases in nutrient concentrations due to anthropogenic activities (i.e., cultural eutrophication; Rissman & Carpenter, 2015). While rapid increases in nutrients can contribute to a number of water‐quality problems (e.g., anoxia, loss of biodiversity, cyanobacterial blooms), these nutrients can also facilitate the abundance of primary producers which can positively affect primary consumers (Baker et al., 2016; Boone & James, 2003; Relyea & Diecks, 2008). Theory suggests that pesticide tolerance of aquatic invertebrates should increase under conditions of nutrient saturation, as organisms have more resources to allocate toward detoxifying environmental contaminants (Jager, Crommentuijn, Gestel, & Kooijman, 2004; Liess, Foit, Knillmann, Schäfer, & Liess, 2016; Pieters et al., 2006). However, despite these theoretical predictions, as well as the common co‐occurrence of pesticide contamination and nutrient enrichment in natural systems, empirical studies investigating how nutrient variability influences pesticide tolerance in non‐target organisms are limited.

*Daphnia pulex*, a common zooplankton species, are useful models for investigating the influence of resource availability on tolerance to pesticides. *Daphnia pulex *are widespread throughout the globe and can inhabit ponds or wetlands located near agricultural activities (Bendis & Relyea, 2014; Declerck et al., 2006). Given their high sensitivities to most chemicals and rapid generation time, *D. pulex *are commonly used in ecotoxicological tests to assess chemical risk in aquatic systems (Newman, 2010). Further, resource levels can be manipulated in the laboratory by varying the algal cell density fed to *D. pulex *(Barry et al., 1995; Pereira & Gonçalves, 2007; Sterner, Hagemeier, Smith, & Smith, 1993). Additionally, *D. pulex *have been used extensively to examine phenotypic plasticity to other stressors, such as predation, which provides a solid foundation for exploring questions related to plasticity to pesticides (Petrusek, Tollrian, Schwenk, Haas, & Laforsch, 2009; Rozenberg et al., 2015; Scheiner & Berrigan, 1998; Tollrian, 1993).

Therefore, using *D. pulex* as our model, we investigated the effects of low and high levels of resources (i.e., algal cell density of *Scenedesmus acutus*) on tolerance to pesticides. Specifically, we asked the following questions: (a) Does resource availability affect baseline tolerance of *D. pulex* to pesticides? (b) Are *D. pulex *able to induce tolerance to pesticides via phenotypic plasticity? (c) Does resource availability influence the ability for *D. pulex* to induce tolerance? We hypothesized that (a) *D. pulex* reared in high resource environments will have higher baseline tolerance than those reared in low resource environments, (b) *D. pulex *reared in environments with sublethal concentrations of pesticides will be able to induce increased tolerance to lethal concentrations of pesticides later in life via phenotypic plasticity, and (c) *D. pulex* reared in high resource environments will induce a greater increase in tolerance compared to those reared in low resource environments.

## 2 MATERIALS AND METHODS

### 2.1 Insecticide background

The insecticide carbaryl (Sevin© 22.5% active ingredient; CAS 63‐25‐2) is an acetylcholine esterase (AChE) inhibiting carbamate insecticide that is used for both home and commercial agriculture application within the United States (Grube et al., 2011). The half‐life of carbaryl is 10 days at a pH of 7, and the maximum concentration detected in aquatic systems is 33.5 µg/L (U.S. EPA, 2012). Similar to other pesticides, carbaryl is able to enter ponds through aerial drift or runoff (Gilliom, 2007). Furthermore, carbaryl has previously been shown to decrease total available energy (i.e., energy associated with respiration, reproduction, and growth) by 26% in *Daphnia magna *(Jeon, Kretschmann, Escher, & Hollender, 2013).

### 2.2 Algal husbandry

We cultured *Scenedesmus acutus *using COMBO water, a medium that supports algal growth (Kilham, Kreeger, Lynn, Goulden, & Herrera, 1998). We autoclaved two, 2‐L glass Erlenmeyer flasks each with 1.2 L of COMBO water and 1.2 ml of algal trace elements (ATE) to sterilize the medium and equipment prior to culturing. We then flooded a petri dish containing *S. acutus *cultures (Jeyasingh Lab in Oklahoma State University) with 5 ml of sterilized COMBO water and added 2.5 ml aliquots of the solution into each of the 2‐L Erlenmeyer flasks. We sealed each flask with a rubber stopper and bubbled air into the mixture using sterilized air stones. We allowed algae to grow for 10 days at 25°C on a 12:12 light–dark cycle in a biosafety cabinet. After the 10 days of growth, the bubbled air supply was shut off, and the algae were allowed to settle for 24 hr. After 24 hr, the excess COMBO water was decanted from the flasks, and the remaining concentrated algae was placed in a sterile 1‐L glass jar and stored at 4°C.

### 2.3 Daphnia husbandry

Past studies have shown that pesticide tolerance in *D. pulex *can vary depending on the population's historical exposure to pesticides (Bendis & Relyea, 2014; Jansen, Coors, Stoks, & Meester, 2011; Jansen, De Meester, Cielen, Buser, & Stoks, 2011). As such, we purchased *D. pulex* from Carolina Biological Supply company's lab stock instead of collecting *D. pulex *from wild populations to reduce the likelihood that *D. pulex* used in the experiment were previously exposed to pesticides. Because our experiment aimed to investigate the interaction between herbivore and algae, we conducted all *D. pulex *husbandry and experiments using a COMBO medium that could simultaneously support both the herbivore and algae (Kilham et al., 1998). To obtain animals for the experiments, we haphazardly selected 60 individuals and cultivated clonal cultures of each individual for four generations until we had at least 200 24‐hr‐old fourth generation (G_4_) *D. pulex*. All *D. pulex *from G_1–3 _generations were fed *S. acutus *from our stock solution *ad libitum* *.*

### 2.4 Experimental setup

#### 2.4.1 Part 1—early environment

**Figure 1:**
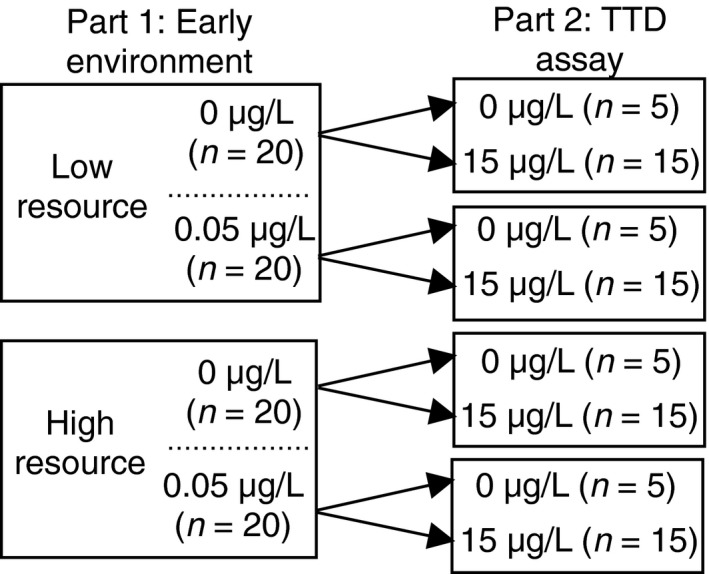
Two‐part experimental design. Part 1 consisted of four treatment solutions containing 0 µg/L carbaryl + low resources, 0.05 µg/L carbaryl + low resources, 0 µg/L carbaryl + high resources, and 0.05 µg/L carbaryl + high resources. During part 2, we initiated the TTD assay by exposing *D. pulex *from each of the four Part 1 treatments to either a control or lethal concentration of carbaryl (15 µg/L)

We filled 50 ml beakers with 45 ml of the four treatment solutions (0 µg/L carbaryl + low resources, 0.05 µg/L carbaryl + low resources, 0 µg/L carbaryl + high resources, 0.05 µg/L carbaryl + high resources; Figure 1). To create these four treatment solutions, we first created two low and two high resource solutions in 1‐L glass jars filled with 800 ml of the treatment solution. The low and high resource solutions contained on average (±standard error) 1,375 ± 396 algal cells/ml and 12,700 ± 550 algal cells/ml, respectively. The algal concentration for the low and high resource treatments were measured by taking five samples from each treatment and counting the number of cells in each sample using a hemocytometer (Sterner, 1993; Sterner et al., 1993).

Next, to create the sublethal carbaryl treatments, we first prepared a 240 µg/L stock solution by adding 2 µl of a 2.39 × 10^8^ µg/L commercial grade carbaryl solution to 2 L of COMBO water. Then we added 208 µl of the stock solution to one of the 1‐L glass jars with the low resource treatment and one of the high resource treatment jars to create a low and high resource treatment that contained 0.05 µg/L of carbaryl, respectively. For the low and high resource treatment that contained 0 µg/L of carbaryl, we mock dosed the remaining low resource and high resource treatment jar with 208 µl of COMBO water.

We then added 45 ml of each treatment solution to their respective experimental units (50‐ml glass beaker). We replicated each of the four treatments 20 times for a total of 80 experimental units. After filling each of the 80 experimental units with their respective solution, we haphazardly transferred a single 24‐hr‐old G_4_ *D. pulex* from a mixture of all available 24‐hr‐old G_4_ *D. pulex* into each of the experimental 50 ml beakers. To prevent cross‐contamination, we used a separate plastic transfer pipette for each treatment. Beakers were haphazardly ordered and held for 5 days at 25°C on a 12:12 light–dark cycle.

#### 2.4.2 Part 2—Time to death (TTD) assay

After 5 days in the early environmental condition treatments, we initiated the TTD assay (Figure 1) by exposing *D. pulex *from each of the four Part 1 treatments to either a control (*n* = 5) or lethal (*n* = 15) concentration of carbaryl (15 µg/L). To create the lethal concentration of carbaryl, we added 281 ml of the 240 µg/L stock solution to 4.5 L of COMBO water. To create the control treatment, we mock dosed 4.5 L of COMBO water with 281 ml of COMBO water. For the TTD assay, we transferred all *D. pulex* to new 50 ml glass beakers filled with 45 ml of the control or lethal carbaryl solution using a transfer pipette. Experimental units were haphazardly organized and held at a constant temperature of 25°C on a 12:12 light–dark cycle. Following standard toxicity assay protocol (Newman, 2010), the individuals were not fed during the TTD assay. To determine time to death of each individual, we conducted hourly checks for the first 12 hr and every 4 hr until 72 hr. To determine mortality, *D. pulex *were observed under a dissecting microscope (Olympus SZ), and mortality was defined as the lack of a heartbeat. To account for any potential variation in *Daphnia* body size across the treatments, following the experiment, we measured the length (top of the carapace to end of tail spine) of the five individuals not exposed to lethal concentrations of carbaryl in the TTD assay from each treatment (0 µg/L carbaryl + low resource; 0 µg/L carbaryl + high resource; 0.05 µg/L carbaryl + low resource; 0.05 µg/L carbaryl + high resource).

### 2.5 Insecticide testing

To confirm the concentrations of carbaryl used in this study, we replicated the dosing procedure and collected a 1‐L sample of the sublethal 0.05 µg/L carbaryl + low resources and 0.05 µg/L carbaryl + high resources treatments during the Part 1 procedures and a 1‐L sample of the lethal 15 µg/L carbaryl during the Part 2 procedures. Because we used COMBO water as the control for both Part 1 and 2, we collected a single 1‐L sample from this source to be tested. All samples were analyzed using ultra‐performance liquid chromatography–tandem mass spectrometry (UPLC‐MS/MS) at the University of Connecticut's Center for Environmental Sciences and Engineering (Storrs, CT). In Phase 1 of the experiment (sublethal exposure), actual concentrations for the 0.05 µg/L carbaryl + low resources and 0.05 µg/L carbaryl + high resources treatments were 0.05 µg/L and 0.04 µg/L carbaryl, respectively (reporting limit = 0.02 µg/L). For Phase 2 of the experiment (lethal exposure treatment), the actual concentration for the 15 µg/L carbaryl treatment was 7.14 µg/L (reporting limit = 0.02 µg/L). We note that the actual concentration of the lethal treatment detected was 52% lower than expected. Despite this lower actual concentration, the design of the experiment was not affected. All animals assigned to the Phase 2 lethal treatment were still exposed to identical lethal conditions (7.14 µg/L of carbaryl) for the TTD assay. Furthermore, studies have found concentrations below 7 µg/L carbaryl to be acutely toxic to *D. pulex* (Eignor, 2012). For consistency, we will refer to the concentrations for the remainder of the paper as either sublethal or lethal. Finally, no pesticides were detected in the control sample.

### 2.6 Statistical analysis

Using an ANOVA, we found no effect of resource (*F*_1,20_ = 2.6; *p* = 0.13), early pesticide exposure (*F*_1,20_ = 3.5; *p* = 0.08), or a pesticide*resource interaction (*F*_1,20_ = 0.6; *p* = 0.45) on *Daphnia* size; therefore, we did not include *D. pulex *size as a covariate in our analyses. To investigate the effects of early pesticide exposure and resource availability on *D. pulex*, we conducted a single Wilcoxon–Gehan D test comparing survival curves of *D. pulex *exposed to each of the treatments (SPSS 21; Pyke & Thompson, 1986; Hoverman, Gray, & Miller, 2010). To address our three questions, we focused on three pairwise comparisons. First, to address whether resource availability affects the baseline tolerance of *D. pulex* to pesticides, we compared survival curves of *D. pulex *from the high versus low resource treatments that were not previously exposed to pesticides. Next, to determine whether *D. pulex *are able to induce tolerance to pesticides, we compared survival curves of individuals not exposed to carbaryl early in life with individuals that were exposed to the sublethal dose of carbaryl for *D. pulex* reared in the low resource treatment and then for those reared in the high resource treatment.

We also examined the effects of early pesticide exposure, resource treatment, and their interaction on the average time to death of *D. pulex, *using a generalized linear model (GENLIN SPSS 21) with a Poisson distribution and an identity function*. *For all significant interactions, we conducted planned contrasts (Sequential Bonferroni) to investigate the drivers of the interaction (EMMEANS SPSS 21). Because the result of the GLM analysis on average TTD was similar to the survival analysis comparing survival curves, we only report the results of the survival analysis. We report the survival analysis results because the survival curves provide more detailed information about when individuals experienced mortality relative to other individuals compared to the GLM analysis on average TTD, which only provides information about mortality at a particular snapshot in time (72 hr). The results of the GLM are reported in the Supporting Information.

## 3 RESULTS

**Figure 2:**
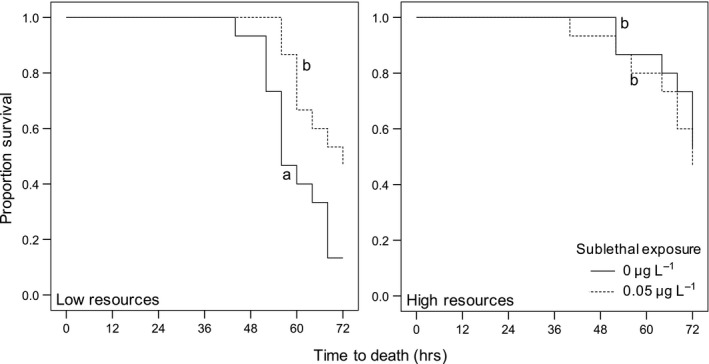
Survival curves of *D. pulex *in low and high resource treatments that were exposed to no carbaryl (0 µg/L) or a sublethal concentration of carbaryl (0.05 µg/L) early in life. Survival curves with different letters represent curves that significantly differ (*p* < 0.05)

We found a significant overall effect of the treatments on the survival curves of *D. pulex *(*G* = 8.8, *p* = 0.032; Figure 2). To address our first question of whether resource availability influences baseline tolerance to pesticides, we compared the survival curves of *D. pulex *raised in the high resource versus low resource treatments that were not previously exposed to carbaryl. We found that *D. pulex *raised in high resources and not exposed to carbaryl had higher baseline tolerance than individuals raised in low resources (*p* = 0.008; Figure 2).

To determine whether *D. pulex* can induce increased tolerance to pesticides and whether resource availability influences the ability to induce tolerance, we compared the survival curves of individuals exposed to no carbaryl early in life versus individuals exposed to sublethal carbaryl within each resource treatment*. *We found that only *D. pulex *from the low resource treatment that were exposed to a sublethal dose early in life were significantly more tolerant to a lethal concentration of carbaryl later in life than individuals not exposed to carbaryl early in life (*p* = 0.016; Figure 2). In contrast, for *D. pulex *in the high resource treatment, we found no evidence of induced tolerance as survival curves of individuals exposed to a sublethal dose early in life did not differ compared to individuals that were not exposed to carbaryl (*p* = 0.933; Figure 2). Finally, we observed 100% survival of the individuals not exposed to lethal concentrations of carbaryl in the TTD assay from each Phase 1 treatment (0 µg/L carbaryl + low resource; 0 µg/L carbaryl + high resource; 0.05 µg/L carbaryl + low resource; 0.05 µg/L carbaryl + high resource).

## 4 DISCUSSION

Understanding whether and how organisms respond to rapidly changing environments initiated by anthropogenic activities will become increasingly important as human populations continue to grow. In this study, we found that a single clone (genotype) of laboratory *D. pulex* reared in high resource environments had higher baseline tolerance to carbaryl compared to those reared in low resource environments. We also discovered the first evidence that *D. pulex *are capable of rapidly inducing tolerance to pesticides via phenotypic plasticity. However, plasticity to carbaryl was context dependent; only *D. pulex* reared in low resource environments induced increased tolerance. Interestingly, though *D. pulex* reared in low resource environments had lower baseline tolerance, individuals exposed to low concentrations of pesticides early in life were able to induce similar tolerances to carbaryl as those reared in the high resource treatment. While this study was conducted on a single clone of laboratory *D. pulex*, and thus comes with limitations, this work suggests that phenotypic plasticity and enhanced resource availability may both be mechanisms allowing *D. pulex *to persist in environments contaminated by carbaryl and consideration of resource variability is imperative to understanding tolerance to pesticides.

Models predict that *Daphnia *reared in low resource environments should be more susceptible to pesticides compared to those reared in high resource environments because fewer resources are available to allocate toward chemical detoxification (English & Uller, 2016; Jager et al., 2004; Pieters et al., 2006). Indeed, our results demonstrate that the average time to death of *D. pulex *reared in the low resource treatment was 16% earlier than individuals reared in the high resource treatment. These results are consistent with previous work which found that *D. magna *and *D. longispina* reared in low resource environments were less tolerant to another carbamate insecticide, methomyl, compared to those reared in high resource environments (Pereira & Gonçalves, 2007). Similarly, in mosquitoes, individuals with resistant phenotypes that were fed multiple times, remained tolerant to insecticides across time whereas those that were fed once lost insecticide tolerance across time (Oliver & Brooke, 2014). The specific mechanisms allowing *D. pulex* to tolerate carbaryl are beyond the scope of our study, but previous work has shown that an upregulation of AChE is one mechanism allowing for *Daphnia *to overcome the toxic effects of carbaryl (Barata, Solayan, & Porte, 2004). While synthesizing new AChE may allow individuals to persist when exposed to carbaryl, upregulating the production of AChE is energetically costly (Jeon et al., 2013). Thus, it is possible that in our study, access to high resources may be allowing *D. pulex* to overcome the energetic costs associated with detoxification, thereby buffering individuals from the toxic effects of carbaryl.

For organisms without high baseline tolerance, phenotypic plasticity may be an alternative mechanism to rapidly respond to contaminants in the environment (Benson & Birge, 1985). We found that *D. pulex* in the low resource environments were able to induce carbaryl tolerance following exposure to low concentrations of the pesticide early in life. Notably, *D. pulex *from low resource environments were able to induce tolerances that matched tolerances of animals reared in high resource environments (Supporting Information). *Daphnia* are well known for their ability to induce adaptive phenotypes in response to changing environmental conditions (i.e., predator‐induced defenses; Petrusek et al., 2009) and have also been shown to respond plastically to heavy metals (e.g., cadmium; Stuhlbacher, Bradley, Naylor, & Calow, 1992). However, this is the first study to show that *Daphnia *plasticity also extends to pesticides. While the mechanism allowing *D. pulex *to induce carbaryl tolerance is not yet known, in wood frogs, individuals that induced tolerance had higher levels of AChE (Hua et al., 2013). In killifish, individuals with inducible tolerance had increased activity of cytochrome P450 1A (CYP1A), which increased the ability to metabolize carbaryl (Oziolor et al., 2017). Therefore, early exposure to sublethal carbaryl may have induced *D. pulex *to upregulate and accumulate AChE protecting them from later exposures (Hua et al., 2013). Alternatively, *D. pulex* may have induced increased cytochrome P450‐ mediated xenobiotic metabolism activity, increasing their ability to metabolize carbaryl (Oziolor et al., 2017). As human activities continue to encroach upon natural systems, considering the role of plasticity in allowing wild populations to respond to rapidly changing conditions and identifying the mechanisms driving these rapid plastic responses has broad conservation implications.

Previous studies have demonstrated that exposure to sublethal concentrations of pesticides early in development can lead to induced tolerance to pesticides later in life (Hua et al., 2015; Jones & Relyea, 2015; Poupardin et al., 2008). However, it is not known whether resource availability alters the ability for organisms to induce tolerance to pesticides. Similar to baseline tolerance, we predicted that individuals reared in high resource environments would also be more likely to induce tolerance to carbaryl compared to individuals reared in low resource environments (Barry et al., 1995; Pereira & Gonçalves, 2007). However, in our study, individuals reared in high resource environments that were also exposed to a sublethal concentration of carbaryl early in life did not induce increased tolerance. Past studies demonstrate that there is a physiological upper limit of *D. pulex *tolerance to AChE‐inhibiting insecticides at >50% inhibition of AChE (Barata et al., 2004). Thus, one possibility for why we did not detect induced tolerance is that *D. pulex *in the high resource treatment were already at the upper limit of carbaryl tolerance. Future work might consider multiple *D. pulex *populations that vary in their upper limits of tolerance. Additionally, due to the higher density of algae in the high food treatment, it is possible that sorption of carbaryl to algae may have prevented the direct exposure of *D. pulex *to carbaryl during the sublethal exposure phase. Future studies should consider assessing the concentrations of carbaryl within both the individual *D. pulex* and the algae. Finally, it is important to note that the low concentration (0.05 µ/L) of carbaryl used in this study likely had no effect on algal densities of *S. acutus*. Indeed, previous research observed no effect of carbaryl at 0.2 and 0.5 mg/L toward *Scenedesmus quadricauada* and *Scenedesmus obliquus*, respectively (Ma et al., 2006), suggesting our resource levels remained constant throughout Phase 1 of our study. As modern agricultural practices continue to introduce synthetic pesticides and nutrient‐rich fertilizers into aquatic systems, consideration of how altered resource availability limits or facilitates pesticide tolerance is necessary.

The discovery that *D. pulex *are capable of plastic responses to pesticides, combined with the fast generation time of *D. pulex *and the relative ease of manipulating environmental conditions, further underscores the utility of *D. pulex *as a model organism for evaluating the role of plasticity in allowing organisms to rapidly respond to anthropogenic chemicals. Additionally*,* due to their relative sensitivity to most contaminants, *D. pulex *are common tools for toxicity assays and risk assessments. However, in nature, *Daphnia *are commonly exposed to low levels of contaminants that could cause them to induce tolerance (Stone et al., 2014). Thus, organisms can rapidly become tolerant to contaminants and overlooking the potential for inducible tolerance may lead to inaccurate assessments of chemical toxicity. Additionally, pre‐exposure to one toxicant could also trigger higher or lower tolerance toward another toxicant later in life (Ashauer, O'Connor, & Escher, 2017). Therefore, future studies should consider whether and how plasticity to pesticides could influence toxicity assays and ultimately the risk assessment process.

Finally, the ability to rapidly respond to pesticides via plasticity may also have broad ecological implications as *D. pulex* contribute major functions to aquatic communities including nutrient cycling and acting as consumers and prey (Boone & James, 2003; Fleeger, Carman, & Nisbet, 2003; Hanazato, 1998, 2001; Relyea & Diecks, 2008; Rohr & Crumrine, 2005). Pesticides and excess fertilizer runoff can indirectly initiate harmful algal blooms that have negative cascading effects on pond communities (Boone & James, 2003; Fleeger et al., 2003; Relyea & Diecks, 2008; Rissman & Carpenter, 2015; Rohr & Crumrine, 2005). Consumers of phytoplankton, such as *D. pulex*, are important consumers that can help to limit negative community effects of algal blooms initiated by pesticide contamination and cultural eutrophication. Therefore, the ability to induce tolerance not only allows *D. pulex* to persist when faced with pesticides but it may also protect pond communities from the negative effects of pesticides by limiting the negative effects of algal blooms (Bendis & Relyea, 2016). Thus, future studies should consider how inducible tolerance to pesticides influence community interactions.

While we believe this study yields important and novel insight into the occurrence of inducible tolerance in *Daphnia*, it is important to note that it was conducted on a single clone (genotype) of laboratory *D. pulex*, and thus our ability to generalize is limited. One limitation of using a single genotype is that we are unable to predict how widespread this phenomenon is. Other studies have demonstrated that there is variation in both pesticide tolerance and associated responses to resource limitation across *Daphnia* genotypes (Pereira & Gonçalves, 2007; Pereira, Mendes, & Gonçalves, 2007); therefore, incorporating genetic variation into future studies should be a priority. Additionally, there may be limitations given that we used a laboratory strain of *D. pulex* rather than a strain collected from the field. Previous research has shown that *Daphnia* require only three generations to remove any variation due to environmental effects (Bendis & Relyea, 2014). Because our cultures were raised in a stock center, and later raised for four generations in our laboratory, it is likely that there were no remaining environmental or maternal effects related to historical pesticide exposure. Given this, future research should consider how naturally occurring populations of *D. pulex* with varying historical pesticide exposure differ in their inducibility to pesticides.

To sum, we demonstrated that *D. pulex *reared in high resource environments had higher baseline tolerance to carbaryl compared to *D. pulex *reared in low resource environments. We also found the first evidence that *D. pulex *are capable of rapidly inducing tolerance to pesticides via phenotypic plasticity. However, contrary to our predictions, *D. pulex *reared in high resource environments did not induce increased tolerance. Instead, only *D. pulex *reared in low resources were able to induce tolerance. Collectively, this study supports the increasing evidence suggesting that inducible tolerance is a widespread phenomenon across different taxa and pollutant types and illustrates the importance of considering environmentally relevant conditions (e.g., resource availability) when making predictions about how organisms may respond to rapidly changing conditions in nature.

## CONFLICT OF INTEREST

None declared.

## AUTHORS CONTRIBUTION

V. P. Wuerthner, G. A. Meindl and J. Hua conceived the ideas, designed methodology, analyzed the data, and contributed critically to the drafts. All authors collected the data and gave final approval for publication.

## Data Availability

A copy of the data will be archived using the DRYAD international repository (http://www.datadryad.org/).

## References

[ece34807-bib-0001] Ashauer, R. , O'Connor, I. , & Escher, B. I. (2017). Toxic mixtures in time—the sequence makes the poison. Environmental Science and Technology, 51, 3084–3092. 10.1021/acs.est.6b06163 28177231

[ece34807-bib-0002] Baker, L. F. , Mudge, J. F. , Thompson, D. G. , Houlahan, J. E. , & Kidd, K. A. (2016). The combined influence of two agricultural contaminants on natural communities of phytoplankton and zooplankton. Ecotoxicology, 25, 1021–1032. 10.1007/s10646-016-1659-1 27112456

[ece34807-bib-0003] Barata, C. , Solayan, A. , & Porte, C. (2004). Role of B‐esterases in assessing toxicity of organophosphorus (chlorpyrifos, malathion) and carbamate (carbofuran) pesticides to *Daphnia magna* . Aquatic Toxicology, 66, 125–139. 15036868 10.1016/j.aquatox.2003.07.004

[ece34807-bib-0004] Barry, M. J. , Logan, D. C. , Ahokas, J. T. , & Holdway, D. A. (1995). Effect of algal food concentration on toxicity of two agricultural pesticides to *Daphnia carinata* . Ecotoxicology and Environmental Safety, 32, 273–279. 10.1006/eesa.1995.1114 8964255

[ece34807-bib-0005] Bendis, R. J. , & Relyea, R. A. (2014). Living on the edge: Populations of two zooplankton species living closer to agricultural fields are more resistant to a common insecticide. Environmental Toxicology and Chemistry, 33, 2835–2841. 10.1002/etc.2749 25220688

[ece34807-bib-0006] Bendis, R. J. , & Relyea, R. A. (2016). If you see one, have you seen them all?: Community‐wide effects of insecticide cross‐resistance in zooplankton populations near and far from agriculture. Environmental Pollution, 1987(215), 234–246. 10.1016/j.envpol.2016.05.020 27208756

[ece34807-bib-0007] Benson, W. H. , & Birge, W. J. (1985). Heavy metal tolerance and metallothionein induction in fathead minnows: Results from field and laboratory investigations. Environmental Toxicology and Chemistry, 4, 209–217. 10.1002/etc.5620040211

[ece34807-bib-0008] Berg, M. P. , Kiers, E. T. , Driessen, G. , Van Der Heijden, M. , Kooi, B. W. , Kuenen, F. , … Ellers, J. (2010). Adapt or disperse: Understanding species persistence in a changing world. Global Change Biology, 16, 587–598. 10.1111/j.1365-2486.2009.02014.x

[ece34807-bib-0009] Blaustein, A. R. , Han, B. A. , Relyea, R. A. , Johnson, P. T. J. , Buck, J. C. , Gervasi, S. S. , & Kats, L. B. (2011). The complexity of amphibian population declines: Understanding the role of cofactors in driving amphibian losses. Annals of the New York Academy of Sciences, 1223, 108–119. 10.1111/j.1749-6632.2010.05909.x 21449968

[ece34807-bib-0010] Boone, M. D. , & James, S. M. (2003). Interactions of an insecticide, herbicide, and natural stressors in amphibian community mesocosms. Ecological Applications, 13, 829–841.

[ece34807-bib-0011] Brausch, J. M. , & Smith, P. N. (2009). Pesticide resistance from historical agricultural chemical exposure in *Thamnocephalus platyurus* (Crustacea: Anostraca). Environmental Pollution, 1987(157), 481–487. 10.1016/j.envpol.2008.09.010 18977573

[ece34807-bib-0012] Cheng, Z. , McCoy, E. L. , & Grewal, P. S. (2014). Water, sediment, and nutrient runoff from urban lawns established on disturbed subsoil or topsoil and managed with inorganic or organic fertilizers. Urban Ecosystem, 17, 277–289.

[ece34807-bib-0013] Chevin, L.‐M. , Lande, R. , & Mace, G. M. (2010). Adaptation, plasticity, and extinction in a changing environment: Towards a predictive theory. PLoS Biology, 8, e1000357. 20463950 10.1371/journal.pbio.1000357 PMC2864732

[ece34807-bib-0014] Coors, A. , & De Meester, L. (2008). Synergistic, antagonistic and additive effects of multiple stressors: Predation threat, parasitism and pesticide exposure in *Daphnia magna* . Journal of Applied Ecology, 45, 1820–1828.

[ece34807-bib-0015] Cothran, R. D. , Brown, J. M. , & Relyea, R. A. (2013). Proximity to agriculture is correlated with pesticide tolerance: Evidence for the evolution of amphibian resistance to modern pesticides. Evolutionary Applications, 6, 832–841. 10.1111/eva.12069 29387169 PMC5779125

[ece34807-bib-0016] Crispo, E. , DiBattista, D. J. , Correa, C. , Thirbert‐Plante, X. , McKellar, A. E. , Schwartz, A. K. , … Henry, A. P. (2010). The evolution of phenotypic plasticity in response to anthropogenic disturbance. Evolutionary Ecology Research, 12, 47–66.

[ece34807-bib-0017] Declerck, S. , De Bie, T. , Ercken, D. , Hampel, H. , Schrijvers, S. , Van Wichelen, J. , … Martens, K. (2006). Ecological characteristics of small farmland ponds: Associations with land use practices at multiple spatial scales. Biological Conservation, 131, 523–532. 10.1016/j.biocon.2006.02.024

[ece34807-bib-0018] Eignor, D. (2012). Aquatic life ambient water quality criteria for carbaryl (EPA‐820‐R‐12‐007). Washington, DC: Office of Water, Science and Technology.

[ece34807-bib-0019] English, S. , & Uller, T. (2016). Does early‐life diet affect longevity? a meta‐analysis across experimental studies. Biology Letters, 12, 20160291 10.1098/rsbl.2016.0291 27601722 PMC5046921

[ece34807-bib-0020] Fleeger, J. W. , Carman, K. R. , & Nisbet, R. M. (2003). Indirect effects of contaminants in aquatic ecosystems. Science of the Total Environment, 317, 207–233. 10.1016/S0048-9697(03)00141-4 14630423

[ece34807-bib-0021] Georghiou, G. (1990). Overview of insecticide resistance InLeBaron H. M., Moberg W. K., & Green M. B. (Eds.), Managing resistance to agrochemicals (pp. 18–41). Washington, DC: American Chemical Society.

[ece34807-bib-0022] Gilliom, R. J. (2007). Pesticides in U.S. streams and groundwater. Environmental Science and Technology, 41, 3408–3414. 10.1021/es072531u 17547156

[ece34807-bib-0023] Grube, A. , Donaldson, D. , Kiely, T. , & Wu, L. (2011). Pesticides industry sales and usage: 2006 and 2007 market estimates. Biological and Economic Analysis Division . Office of Pesticide Programs. Office of Chemical Safety and Pollution Prevention. U.S. Environmental Protection Agency, Washington, DC . Retrieved from https://www.epa.gov/sites/production/files/2015-10/documents/market_estimates2007.pdf

[ece34807-bib-0025] Hanazato, T. (1998). Response of a zooplankton community to insecticide application in experimental ponds: A review and the implications of the effects of chemicals on the structure and functioning of freshwater communities. Environmental Pollution, 101, 361–373. 10.1016/S0269-7491(98)00053-0

[ece34807-bib-0026] Hanazato, T. (2001). Pesticide effects on freshwater zooplankton: An ecological perspective. Environmental Pollution, 112, 1–10. 10.1016/S0269-7491(00)00110-X 11202648

[ece34807-bib-0027] Hoffmann, A. A. , & Sgrò, C. M. (2011). Climate change and evolutionary adaptation. Nature, 470, 479–485. 10.1038/nature09670 21350480

[ece34807-bib-0028] Hoverman, J. T. , Gray, M. J. , & Miller, D. L. (2010). Anuran susceptibilities to ranaviruses: Role of species identity, exposure route, and a novel virus isolate. Diseases of Aquatic Organisms, 89, 97–107. 20402227 10.3354/dao02200

[ece34807-bib-0029] Hua, J. , Jones, D. K. , Mattes, B. M. , Cothran, R. D. , Relyea, R. A. , & Hoverman, J. T. (2015). The contribution of phenotypic plasticity to the evolution of insecticide tolerance in amphibian populations. Evolutionary Applications, 8, 586–596. 10.1111/eva.12267 26136824 PMC4479514

[ece34807-bib-0030] Hua, J. , Morehouse, N. I. , & Relyea, R. (2013). Pesticide tolerance in amphibians: Induced tolerance in susceptible populations, constitutive tolerance in tolerant populations. Evolutionary Applications, 6, 1028–1040. 24187585 10.1111/eva.12083 PMC3804236

[ece34807-bib-0031] Jager, T. , Crommentuijn, T. , van Gestel, C. A. M. , & Kooijman, S. A. L. M. (2004). Simultaneous modeling of multiple end points in life‐cycle toxicity tests. Environmental Science and Technology, 38, 2894–2900. 10.1021/es0352348 15212265

[ece34807-bib-0032] Jansen, M. , Coors, A. , Stoks, R. , & De Meester, L. (2011). Evolutionary ecotoxicology of pesticide resistance: A case study in *Daphnia* . Ecotoxicology, 20, 543–551. 21380529 10.1007/s10646-011-0627-z

[ece34807-bib-0033] Jansen, M. , De Meester, L. , Cielen, A. , Buser, C. C. , & Stoks, R. (2011). The interplay of past and current stress exposure on the water flea *Daphnia* . Functional Ecology, 25, 974–982.

[ece34807-bib-0034] Jeon, J. , Kretschmann, A. , Escher, B. I. , & Hollender, J. (2013). Characterization of acetylcholinesterase inhibition and energy allocation in *Daphnia magna* exposed to carbaryl. Ecotoxicology and Environmental Safety, 98, 28–35. 10.1016/j.ecoenv.2013.09.033 24139064

[ece34807-bib-0035] Jones, D. K. , & Relyea, R. A. (2015). Here today, gone tomorrow: Short‐term retention of pesticide‐induced tolerance in amphibians. Environmental Toxicology and Chemistry, 34, 2295–2301. 25940070 10.1002/etc.3056

[ece34807-bib-0036] Kilham, S. S. , Kreeger, D. A. , Lynn, S. G. , Goulden, C. E. , & Herrera, L. (1998). COMBO: A defined freshwater culture medium for algae and zooplankton. Hydrobiologia, 377, 147–159.

[ece34807-bib-0037] Lawrence, D. , Fiegna, F. , Behrends, V. , Bundy, J. G. , Phillimore, A. B. , Bell, T. , & Barraclough, T. G. (2012). Species interactions alter evolutionary responses to a novel environment. PLoS Biology, 10, e1001330 10.1371/journal.pbio.1001330 22615541 PMC3352820

[ece34807-bib-0038] Liess, M. , Foit, K. , Knillmann, S. , Schäfer, R. B. , & Liess, H.‐D. (2016). Predicting the synergy of multiple stress effects. Scientific Reports, 6, srep32965 10.1038/srep32965 PMC5017025 27609131

[ece34807-bib-0039] Ma, J. , Lu, N. , Qin, W. , Xu, R. , Wang, Y. , & Chen, X. (2006). Differential responses of eight cyanobacterial and green algal species, to carbamate insecticides. Ecotoxicology and Environmental Safety, 63, 268–274. 16677910 10.1016/j.ecoenv.2004.12.002

[ece34807-bib-0040] McKnight, U. S. , Rasmussen, J. J. , Kronvang, B. , Binning, P. J. , & Bjerg, P. L. (2015). Sources, occurrence and predicted aquatic impact of legacy and contemporary pesticides in streams. Environmental Pollution, 200, 64–76. 25697475 10.1016/j.envpol.2015.02.015

[ece34807-bib-0041] Moe, S. J. , De Schamphelaere, K. , Clements, W. H. , Sorensen, M. T. , Van den Brink, P. J. , & Liess, M. (2013). Combined and interactive effects of global climate change and toxicants on populations and communities. Environmental Toxicology and Chemistry, 32, 49–61. 10.1002/etc.2045 23147390 PMC3601420

[ece34807-bib-0042] Newman, M. C. (2010). Fundamentals of ecotoxicology. Boca Raton, FL: CRC Press.

[ece34807-bib-0043] Oliver, S. V. , & Brooke, B. D. (2014). The effect of multiple blood‐feeding on the longevity and insecticide resistant phenotype in the major malaria vector *Anopheles arabiensis* (Diptera: Culicidae). Parasites & Vectors, 7(1), 390. 25150975 10.1186/1756-3305-7-390 PMC4161849

[ece34807-bib-0044] Oziolor, E. M. , Howard, W. , Lavado, R. , & Matson, C. W. (2017). Induced pesticide tolerance results from detoxification pathway priming. Environmental Pollution, 1987(224), 615–621. 10.1016/j.envpol.2017.02.046 28259584

[ece34807-bib-0045] Pereira, J. L. , & Gonçalves, F. (2007). Effects of food availability on the acute and chronic toxicity of the insecticide methomyl to *Daphnia* spp. Science of the Total Environment, 386, 9–20. 10.1016/j.scitotenv.2007.07.040 17727918

[ece34807-bib-0046] Pereira, J. L. , Mendes, C. D. , & Gonçalves, F. (2007). Short‐ and long‐term responses of *Daphnia* spp. to propanil exposures in distinct food supply scenarios. Ecotoxicology and Environmental Safety, 68, 386–396. 10.1016/j.ecoenv.2006.10.012 17150251

[ece34807-bib-0047] Petrusek, A. , Tollrian, R. , Schwenk, K. , Haas, A. , & Laforsch, C. (2009). A “crown of thorns” is an inducible defense that protects *Daphnia* against an ancient predator. Proceedings of the National Academy of Sciences of the United States of America, 106, 2248–2252. 19144929 10.1073/pnas.0808075106 PMC2650140

[ece34807-bib-0048] Pieters, B. J. , Jager, T. , Kraak, M. H. S. , & Admiraal, W. (2006). Modeling responses of *Daphnia* *magna* to pesticide pulse exposure under varying food conditions: Intrinsic versus apparent sensitivity. Ecotoxicology, 15, 601–608. 10.1007/s10646-006-0100-6 17024561

[ece34807-bib-0049] Pigliucci, M. (2001). Phenotypic plasticity: Beyond nature and nurture. Baltimore, MD: Johns Hopkins University Press.

[ece34807-bib-0050] Poupardin, R. , Reynaud, S. , Strode, C. , Ranson, H. , Vontas, J. , & David, J.‐P. (2008). Cross‐induction of detoxification genes by environmental xenobiotics and insecticides in the mosquito *Aedes aegypti*: Impact on larval tolerance to chemical insecticides. Insect Biochemistry and Molecular Biology, 38, 540–551. 10.1016/j.ibmb.2008.01.004 18405832

[ece34807-bib-0051] Puccinelli, E. (2012). How can multiple stressors combine to influence ecosystems and why is it important to address this question? Integrated Environmental Assessment and Management, 8, 201–202. 10.1002/ieam.1250 22184149

[ece34807-bib-0052] Pyke, D. A. , & Thompson, J. N. (1986). Statistical analysis of survival and removal rate experiments. Ecology, 67, 240–245. 10.2307/1938523

[ece34807-bib-0053] Relyea, R. A. , & Diecks, N. (2008). An unforeseen chain of events: Lethal effects of pesticides on frogs at sublethal concentrations. Ecological Applications, 18, 1728–1742. 10.1890/08-0454.1 18839767

[ece34807-bib-0054] Rissman, A. R. , & Carpenter, S. R. (2015). Progress on nonpoint pollution: Barriers & opportunities. Daedalus, 144, 35–47.

[ece34807-bib-0055] Rohr, J. R. , & Crumrine, P. W. (2005). Effects of an herbicide and an insecticide on pond community structure and processes. Ecological Applications, 15, 1135–1147. 10.1890/03-5353

[ece34807-bib-0056] Rozenberg, A. , Parida, M. , Leese, F. , Weiss, L. C. , Tollrian, R. , & Manak, J. R. (2015). Transcriptional profiling of predator‐induced phenotypic plasticity in *Daphnia pulex* . Frontiers in Zoology, 12(1), 18. 26213557 10.1186/s12983-015-0109-x PMC4514973

[ece34807-bib-0057] Scheiner, S. M. , & Berrigan, D. (1998). The genetics of phenotypic plasticity. VIII. The cost of plasticity in *Daphnia pulex* . Evolution, 52, 368–378. 10.2307/2411074 28568340

[ece34807-bib-0058] Schlichting, C. D. (2008). Hidden reaction norms, cryptic genetic variation, and evolvability. Annals of the New York Academy of Sciences, 1133, 187–203. 10.1196/annals.1438.010 18559822

[ece34807-bib-0059] Sterner, R. W. (1993). *Daphnia* growth on varying quality of *Scenedesmus*: Mineral limitation of zooplankton. Ecology, 74, 2351–2360. 10.2307/1939587

[ece34807-bib-0060] Sterner, R. W. , Hagemeier, D. D. , Smith, W. L. , & Smith, R. F. (1993). Phytoplankton nutrient limitation and food quality for *Daphnia* . Limnology and Oceanography, 38, 857–871.

[ece34807-bib-0061] Stone, W. W. , Gilliom, R. J. , & Ryberg, K. R. (2014). Pesticides in U.S. streams and rivers: Occurrence and trends during 1992–2011. Environmental Science and Technology, 48, 11025–11030. 10.1021/es5025367 25209419

[ece34807-bib-0062] Stuhlbacher, A. , Bradley, M. C. , Naylor, C. , & Calow, P. (1992). Induction of cadmium tolerance in two clones of *Daphnia magna straus* . Comparative Biochemistry and Physiology Part C: Comparative Pharmacology, 101, 571–577. 10.1016/0742-8413(92)90088-O 1354135

[ece34807-bib-0063] Tollrian, R. (1993). Neckteeth formation in *Daphnia pulex* as an example of continuous phenotypic plasticity: Morphological effects of *Chaoborus* kairomone concentration and their quantification. Journal of Plankton Research, 15, 1309–1318.

[ece34807-bib-0064] U.S. EPA (2012). Aquatic life ambient water quality criteria for carbaryl.U.S. Environmental Protection Agency Office of Water. Office of Science and Technology. Health and Ecological Criteria Division. Washington, DC. Retrieved from https://www.epa.gov/sites/production/files/2015-08/documents/aquatic_life_ambient_water_quality_criteria_for_carbaryl_-_2012.pdf

[ece34807-bib-0065] West‐Eberhard, M. J. (2003). Developmental plasticity and evolution, 1st ed. Oxford, NY: Oxford University Press.

