# Supplementary material for: Inducible pesticide tolerance in Daphnia pulex influenced by resource availability
Source: Ecol Evol. 2019 Jan 10;9(3):1182–90. doi: 10.1002/ece3.4807 (PMC6374683; doi:10.1002/ece3.4807)
Supplement: Supplementary file 1 [file ECE3-9-1182-s001.docx]

Supplemental material

**Table S1.** The effects of early pesticide exposure, resource treatment, and their interaction on the average time to death of *D. pulex,* using a generalized linear model (GENLIN SPSS 21) with a Poisson distribution and an identity function*.*

|  | *Χ*^2^ | df | P |
| --- | --- | --- | --- |
| Overall | 19.4 | 5 | **0.002** |
| Pesticide treatment | 5.8 | 2 | 0.056 |
| Resource treatment | 5.7 | 1 | **0.017** |
| Pesticide*Resource | 8.5 | 2 | **0.014** |

**Table S2.** Planned contrasts (Sequential Bonferroni) to investigate the significant interaction of pesticide and resource treatments on average time to death of *D. pulex* (EMMEANS SPSS 21).

| Pesticide treatment | Resource treatment | Pesticide treatment | Resource treatment | P-value |
| --- | --- | --- | --- | --- |
| 0 ug L^-1^ | Low | 0 ug L^-1^ | High | **0.003** |
|  |  | 0.05 ug L^-1^ | Low | **0.009** |
|  |  | 0.05 ug L^-1^ | High | **0.04** |
| 0 ug L^-1^ | High | 0.05 ug L^-1^ | Low | 1.0 |
|  |  | 0.05 ug L^-1^ | High | 1.0 |
| 0.05 ug L^-1^ | Low | 0.05 ug L^-1^ | High | 1.0 |

**Table S3.** Pairwise comparisons (Wilcoxon-Gehan D tests) of the survival curves of *D. pulex* from the TTD assay.

| Pesticide treatment | Resource treatment | Pesticide treatment | Resource treatment | P-value |
| --- | --- | --- | --- | --- |
| 0 ug L^-1^ | Low | 0 ug L^-1^ | High | **0.009** |
|  |  | 0.05 ug L^-1^ | Low | **0.016** |
|  |  | 0.05 ug L^-1^ | High | **0.027** |
| 0 ug L^-1^ | High | 0.05 ug L^-1^ | Low | 0.916 |
|  |  | 0.05 ug L^-1^ | High | 0.933 |
| 0.05 ug L^-1^ | Low | 0.05 ug L^-1^ | High | 0.967 |
